# Supplementary material for: Oncologic outcomes after resection of para-aortic lymph node metastasis in left-sided colon and rectal cancer
Source: PLoS One. 2020 Nov 16;15(11):e0241815. doi: 10.1371/journal.pone.0241815 (PMC7668564; doi:10.1371/journal.pone.0241815)
Supplement: S1 Dataset — (PDF) [file pone.0241815.s001.pdf]

No	Age	Sex	Location of tumor	Adjuvant treatment
1	51	Female	Sigmoid colon	No
2	66	Female	Transverse colon	Yes
3	62	Female	Sigmoid colon	No
4	64	Male	Rectum	No
5	64	Female	Rectum	No
6	56	Female	Sigmoid colon	Yes
7	55	Female	Rectum	Yes
8	74	Male	Sigmoid colon	Yes
9	74	Male	Sigmoid colon	Yes
10	63	Male	Rectum	No
11	51	Male	Sigmoid colon	Yes
12	60	Female	Rectum	Yes
13	52	Male	Rectum	No
14	63	Female	Rectum	Yes
15	35	Male	Rectum	Yes
16	65	Male	Rectum	Yes
17	59	Female	Rectum	No
18	72	Male	Rectum	No
19	60	Male	Rectum	Yes
20	54	Male	Rectum	No
21	60	Male	Sigmoid colon	Yes
22	53	Male	Sigmoid colon	Yes
23	46	Male	Sigmoid colon	Yes
24	59	Female	Sigmoid colon	Yes
25	71	Female	Descending colon	Yes
26	63	Female	Sigmoid colon	No
27	50	Female	Rectum	Yes
28	65	Male	Rectum	Yes
29	72	Female	Transverse colon	No

Neoadjuvant chemotherapy	Neoadjuvant radiation therapy
No	No
No	No
No	No
No	No
No	No
No	No
Yes	No
No	No
No	No
No	No
No	No
No	No
No	No
No	No
No	No
No	No
No	No
No	No
No	No
No	No
No	No
No	No
No	No
No	No
No	No
No	No
No	No
No	No
No	No

Adjuvant chemotherapy	Adjuvant chemoradiation therapy	Histology
No	No	Moderately
Yes	No	Moderately
No	No	Moderately
No	No	Moderately
No	No	Moderately
Yes	No	Moderately
No	No	Moderately
Yes	No	Moderately
Yes	No	Moderately
No	No	Moderately
Yes	No	Moderately
Yes	No	Moderately
No	No	Poorly
Yes	No	Well
Yes	No	Poorly
Yes	No	Moderately
No	No	Moderately
No	No	Mucinous
Yes	No	Well
No	No	Moderately
Yes	No	Moderately
Yes	No	Moderately
Yes	No	Mucinous
Yes	No	Poorly
No	Yes	Poorly
No	No	Poorly
Yes	No	Moderately
No	Yes	Poorly
No	No	Moderately

Depth of invasion	pericolic/perirectal lymph node metastasis
pT3	Yes
pT3	Yes
pT4b	Yes
pT4b	No
pT3	Yes
pT4a	Yes
pT3	Yes
pT4a	Yes
pT4a	Yes
pT4a	Yes
pT3	No
pT3	Yes
pT3	Yes
pT3	No
pT4a	Yes
pT3	Yes
pT3	Yes
pT4a	Yes
pT4b	Yes
pT4a	No
pT4a	Yes
pT4a	Yes
pT3	Yes
pT4b	Yes
pT3	Yes
pT4b	No
pT4a	Yes
pT4a	Yes
pT3	No

Intermediate lymph node metastasis	Main lymph node metastasis
Yes	Yes
Yes	No
Yes	No
No	No
No	Yes
No	Yes
No	No
Yes	No
No	Yes
No	No
Yes	No
Yes	Yes
Yes	Yes
Yes	Yes
Yes	Yes
No	No
Yes	No
Yes	Yes
Yes	Yes
Yes	Yes
Yes	Yes
Yes	Yes
Yes	No
Yes	Yes
Yes	Yes
No	No
No	No
Yes	No
No	No

Lateral lymph node metastasis	Number of harvested PALNs
No	11
No	2
No	2
No	1
No	25
No	11
No	2
No	1
No	1
No	1
No	1
No	9
No	2
Yes	4
Yes	71
Yes	7
No	1
Yes	1
Yes	10
No	4
No	10
No	8
No	7
Yes	4
No	4
No	1
No	1
No	4
No	1

Number of metastatic PALNs	Liver metastasis	Lung metastasis
31	No	No
11	No	No
21	No	No
14	No	No
25	No	No
12	No	No
26	Yes	No
3	No	No
2	No	No
12	No	No
13	Yes	No
12	Yes	No
13	No	No
6	No	No
81	Yes	No
21	Yes	No
5	No	No
27	No	No
16	Yes	No
20	Yes	No
11	Yes	No
11	No	No
7	No	No
9	No	No
45	No	No
3	No	No
10	No	No
20	No	No
1	No	No

peritoneal metastasis	Other metastases	Operative procedure
No	No	Open
No	No	Open
No	No	Open
No	No	Open
No	No	Laparoscopic
No	No	Open
No	No	Open
Yes	No	Open
No	No	Open
No	No	Open
No	No	Open
No	No	Open
No	No	Open
No	No	Open
No	No	Open
No	No	Open
No	No	Open
No	No	Open
No	No	Open
Yes	No	Open
No	No	Open
No	No	Open
No	No	Open
Yes	No	Open
No	No	Open
No	No	Open
No	No	Open
No	No	Open
No	No	Open

Operation time	Blood loss	Length of hospital stay	Morbidity
238	491	21	Yes
179	140	15	No
285	1120	47	Yes
282	1898	24	Yes
305	44	10	No
234	350	23	No
158	91	12	No
290	661	50	No
110	20	40	No
214	280	44	No
220	894	28	No
240	410	40	No
180	589	58	Yes
208	493	69	No
645	1814	67	No
640	4900	71	Yes
180	235	35	No
585	2190	85	Yes
460	1350	106	Yes
449	1721	30	No
282	208	33	No
231	314	8	No
259	628	49	No
209	728	22	Yes
295	785	45	Yes
255	596	29	No
248	891	42	No
261	1035	61	No
162	246	49	No

Details of morbidity	Clavien-Dindo classification	Perioperative death
surgical site infection	Ⅲa	No
		No
surgical site infection	Ⅱ	No
Intra-abdominal abcess	Ⅱ	No
		No
		No
		No
		No
		No
		No
		No
		No
Anastomotic leakage	Ⅲb	No
		No
		No
Paralytic ileus	Ⅲa	No
		No
urinary retention	Ⅰ	No
surgical site infection	Ⅱ	No
		No
		No
		No
		No
Atelectasis	Ⅱ	No
Delayed gastric emptying	Ⅱ	No
		No
		No
		No
		No

Outcome	Recurrence	Recurence site_1	Recurence site_2
Death	Yes		
Survive	No		
Survive	Yes		
Survive	Yes		
Survive	Yes		
Death	Yes	Liver	
Survive	Yes	Liver	
Death	Yes		
Survive	Yes		Lung
Death	Yes		
Death	Yes	Liver	
Death	Yes		Lung
Death	Yes		
Death	Yes	Liver	
Death	Yes	Liver	
Death	Yes		Lung
Survive	Yes	Liver	
Death	Yes		
Death	Yes		
Death	Yes	Liver	
Death	Yes	Liver	
Death	No		
Death	No		
Death	Yes		
Death	Yes		
Death	No		
Survive	No		
Death	Yes		
Survive	No		

Recurence site_3	Recurence site_others	follow-up time
	Distant metastasis	1638
	なし	1660
Peritoneum	Distant metastasis	1391
	Local recurrence	1496
	Distant metastasis	2140
	なし	1605
	なし	550
Peritoneum	なし	444
	なし	4717
	Others	1472
	なし	858
	なし	822
	Distant metastasis	802
	Distant metastasis	668
	Distant metastasis	145
	Distant metastasis	1908
	Distant metastasis	4590
	Local recurrence	691
	Others	899
	なし	1120
	なし	487
	なし	791
	なし	204
Peritoneum	Distant metastasis	353
	Bone	688
	なし	45
	なし	6321
Peritoneum	なし	2914
	なし	5274

Recurrence-free survival time
27
1660
544
846
59
63
166
354
1112
286
78
386
537
525
35
670
278
574
605
372
120
791
204
129
628
45
6321
2884
5274
